# Prevalence of tick-transmitted pathogens in cattle reveals that *Theileria parva*, *Babesia bigemina* and *Anaplasma marginale* are endemic in Burundi

**DOI:** 10.1186/s13071-020-04531-2

**Published:** 2021-01-05

**Authors:** Lionel Nyabongo, Esther G. Kanduma, Richard P. Bishop, Eunice Machuka, Alice Njeri, Alain V. Bimenyimana, Canesius Nkundwanayo, David O. Odongo, Roger Pelle

**Affiliations:** 1 School of Biological Sciences, University of Nairobi (UoN), Nairobi, Kenya; 2 Biosciences eastern and central Africa-International Livestock Research Institute (BecA-ILRI) Hub, P.O. Box 30709, Nairobi, Kenya; 3 National Veterinary Laboratory, Bujumbura, Burundi; 4 Department of Biochemistry, School of Medicine, University of Nairobi, Nairobi, Kenya; 5 Veterinary Microbiology and Pathology (VMP), Washington State University, 100 Dairy Road, Pullman, WA 99164 USA; 6 International Livestock Research Institute (ILRI), P.O. Box 30709, Nairobi, Kenya; 7 Programme National pour la Sécurité Alimentaire et le Développement Rural de l’Imbo et du Moso (PNSADR-IM), Bujumbura, Burundi

**Keywords:** Mapping, Tick-borne infections, Agro-ecological zone, Prevalence, *Theileria*, *Anaplasma*, *Babesia*

## Abstract

**Background:**

Tick-borne diseases (TBDs) constitute a major constraint for livestock development in sub-Saharan Africa, with East Coast fever (ECF) being the most devastating TBD of cattle. However, in Burundi, detailed information is lacking on the current prevalence of TBDs and on the associated economic losses from mortality and morbidity in cattle as well as the costs associated with TBD control and treatment. The aim of this study was, therefore, to assess the prevalence and spatial distribution of tick-borne pathogens (TBPs) in cattle across the major agro-ecological zones (AEZs) in Burundi.

**Methods:**

In a cross-sectional study conducted in ten communes spanning the five main AEZs in Burundi, blood samples were taken from 828 cattle from 305 farms between October and December 2017. Evidence of *Theileria parva* infection was assessed by antibody level, measured using a polymorphic immunodominant molecule (PIM) antigen-based enzyme-linked immunosorbent assay (ELISA) and by a *T. parva-*specific p104 gene-based nested PCR. Antibodies against *Theileria mutans* infection were detected using the 32-kDa antigen-based indirect ELISA, while the 200-kDa antigen and the major surface protein 5 (MSP5)-based indirect ELISA were used to detect antibodies against *Babesia bigemina* and *Anaplasma marginale*, respectively.

**Results:**

The prevalence of *T. parva* across the ten communes sampled ranged from 77.5 to 93.1% and from 67.8 to 90.0% based on the ELISA and PCR analysis, respectively. A statistically significant difference in infection was observed between calves and adult cattle; however, *T. parva* infection levels were not significantly associated with sex and breed. The seroprevalence indicating exposure to *T. mutans*, *B. bigemina* and *A. marginale* ranged from 30 to 92.1%, 33.7 to 90% and 50 to 96.2%, respectively. Mixed infections of TBPs were detected in 82.91% of cattle sampled, with 11 different combinations of pathogen species detected .

**Conclusions:**

The findings indicate that *T. parva*,* A. marginale* and* B. bigemina* infections are endemic in Burundi. Knowledge of the spatial distribution of TBPs will facilitate the design of effective targeted strategies to control these diseases. There is a need for further investigations of the distribution of tick vectors and the population structure of TBPs in order to identify the key epidemiological factors contributing to TBD outbreaks in Burundi.
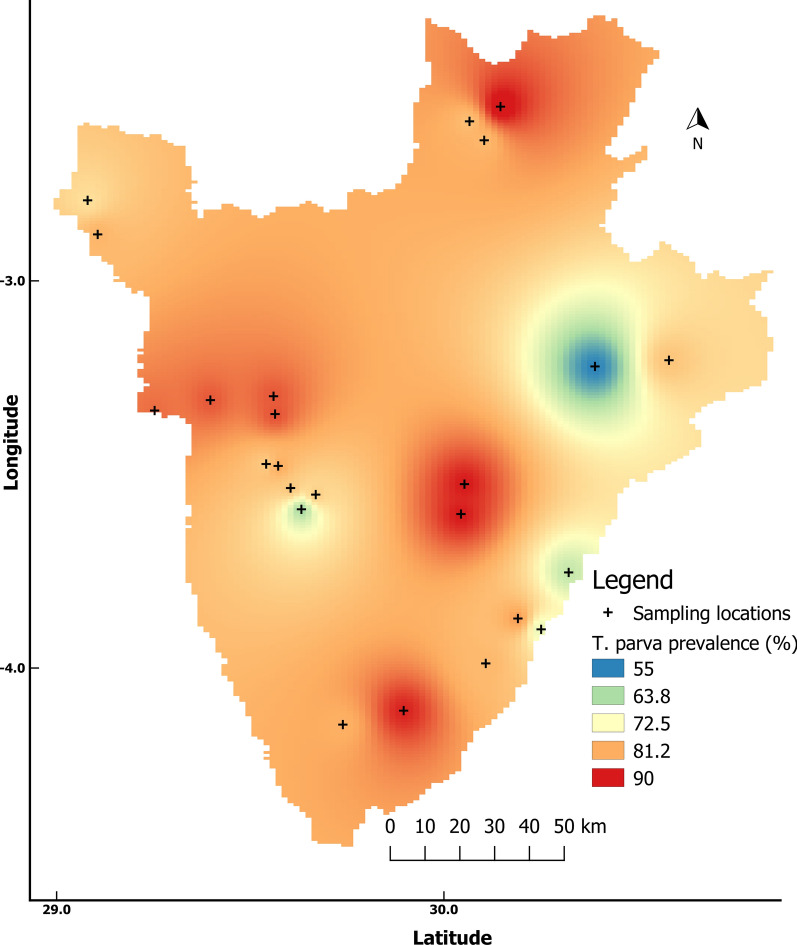

## Background

The livestock sector is a key component of the national development program in Burundi, contributing 4.6% to the gross domestic product, with 90% of the population depending on agriculture and livestock for their livelihood [1]. The cattle population is estimated at 620,000 animals, which are reared across different agro-ecological zones (AEZs) in Burundi [1, 2]. Within the last decade, the Holstein cattle breed has been imported from neighboring countries and distributed among rural farmers in an attempt to improve the productivity of the local breed, Ankole. However, cattle productivity remains low, mainly due to ticks and tick-borne diseases (TBDs) as well as other abiotic factors that constrain livestock production.

TBDs cause considerable economic losses in tropical and subtropical areas of Africa, primarily due to morbidity and mortality in susceptible cattle combined with the costs of treating and controlling TBDs. East Coast fever (ECF), caused by the protozoan parasite *Theileria parva*, is a lymphoproliferative disease of cattle that is transmitted by the brown ear tick *Rhipicephalus appendiculatus*. Animal health reports indicate a high number of clinical cases of ECF in Burundi, with more than 5% of the cattle population affected annually [3]. Earlier studies conducted in Burundi showed a prevalence of ECF ranging between 10.9–29.9% and 48.5–62.8% depending on the testing method, i.e. microscopic examination of blood smears and the indirect fluorescent antibody test (IFAT), respectively [4]. The distribution of ECF has also been found to be correlated with the distribution of the tick vector. Immunization against ECF using the “infection and treatment method” (ITM) involves the inoculation of a live, potentially lethal dose of the parasite and simultaneous treatment with a long-acting formulation of oxytetracycline [5]. Since protection against ECF is sometimes parasite strain specific, combinations or cocktails of stocks that provide significant protection against heterologous challenge in the field have been developed. The most widely used is the trivalent Muguga cocktail which comprises of *T. parva* Muguga, Kiambu 5 and Serengeti-transformed stocks [6]. This cocktail combination is being used extensively in East Africa [7], but is yet to be deployed in Burundi. An ITM using locally derived *T. parva* stocks comprising Gatumba, Gitega and Ngozi isolates was deployed within limited areas of Burundi from 1987, but was terminated in 1993 due to the civil war [8].

Cattle infection with *Anaplasma marginale* and *Babesia bigemina*, the causative agents of bovine anaplasmosis and bovine babesiosis, respectively, was confirmed in Burundi in 1989 using microscopy and serology, with 42–62% of apparently healthy cattle being found to be infected with *A. marginale*. The overall prevalence of *B. bigemina* infection was estimated at 1.3%, with 1.5–3% of infections associated with clinical signs of the disease; the remainder were asymptomatic cases [4]. The distribution of bovine babesiosis due to *B. bigemina* has been found to be closely associated with the presence of *Boophilus decoloratus*, the main tick vector. Although the above data indicate that *T. parva*, *A. marginale* and *B. bigemina* cattle infections are present in Burundi, the information available is now outdated [9]. Moreover, these reports were based on IFAT and microscopic techniques which are not very sensitive. Thus, the current distribution, disease status and prevalence of these pathogens are unknown.

Control strategies for TBDs, either through tick vector control, host immunization or disease treatment, should be based on detailed information relating to the disease epidemiology. The objective of this study was to determine the prevalence of tick-borne pathogens (TBPs) in cattle across different AEZs of Burundi using serological and molecular assays. The data obtained were also used to establish the extent of TBP distribution. This information will guide and inform the design of effective disease control strategies.

## Methods

### Study area

Burundi is a landlocked country of 27,834 square kilometres, located between latitude 2°45′ and 4°28′S and longitudes 28°50′ and 30°53′E. The country is structured in three administrative levels: provinces, communes and collines (district). The overall climate is tropical humid with four main seasons: a major rainy season from February to May; a minor rainy season from September to November; a dry season from June to August; and a minor dry season between December and January. The study was carried out in ten communes located across the five AEZs of Burundi, which are, from west to east: the western plains of Imbo; the slope of the Congo Nile Divide (Mumirwa); the Congo–Nile Divide (CND); the Central Highlands; and the depressions of Kumoso and Bugesera. The AEZs are defined based on (i) monthly temperature (tropic, subtropic), (ii) moisture (humid, sub-humid, arid, semi-arid), (iii) elevation adjusted to sea level (highland/cool and lowland/warm), (iv) landform and (v) soil type [10].

The Imbo zone is the lowland zone of Burundi and is characterized by an average altitude of 700–900 m a.s.l., an average annual rainfall of 1000 mm and an average temperature of between 23 °C and 24.2 °C. The capital city of Bujumbura is located in this area, with the result that there is a high demand for milk. This has led to farmers rearing crossbred and taurine cattle under the auspices of an intensive production system in an effort to increase milk production. The Central Highlands cover 52% of the total surface area of the country and are situated at an altitude of between 1300 and 1500 m a.s.l.; the average temperature ranges from 18 °C to 21 °C and average annual rainfall ranges from 1200 to 1600 mm. The depressions of Kumoso (east) and Bugesera (north) are situated at an altitude of between 1100 and 1400 m a.s.l., with temperatures ranging from 21 °C to 23 °C and an annual rainfall of between 100 and 1300 mm. The depressions cover 16% of the total surface area of the country and have a good coverage of pastures suitable grazing areas. Although the depressions of Bugesera are surrounded by two lakes, namely Lake Cohoha and Lake Rweru, the area is characterized by a lack of water resources for livestock. The CND, a highland area of Burundi situated at an altitude of 1700–2500 m a.s.l., has an average temperature of 21–23 °C, covers 15% of the total surface area of the country and is suitable for livestock grazing, with more than 41% of the country’s cattle reared in this area [1]. The slope of the CND (Mumirwa), located between the Imbo lowlands and the CND, covers 10% of the surface area of the country and is situated at an altitude of between 1000 and 1700 m a.s.l.

The local breed of African cattle, Ankole, is the predominant breed reared across the country under an extensive production system where animals are kept with minor inputs from the farmer. Crossbred cattle characterized by a high dairy potential are kept under semi-intensive management system by approximately 20% of farmers [11]. The intensive dairy farming management system is present in some peri-urban districts where farmers rear improved taurine breeds [12]. The vegetation of the study area has been described in a previous study [1].

### Study design and data collection

A cross-sectional study was conducted in ten selected communes of Burundi between October and December 2017. It was determined that a total of 783 cattle should be sampled to achieve a confidence level of 99% and a margin of error of 3.5% in the analysis of a cattle population of 620,000 heads. In this study, 828 cattle were sampled across the country with a random selection of cattle from each commune. Calves aged < 21 weeks were not sampled since maternal *T. parva* antibodies confound the analysis prior to this age [13]. Blood samples were collected in ethylenediaminetetraacetic acid (EDTA) tubes and vacutainer plain tubes by jugular vein puncture and transported in cool boxes to the laboratory where the vacutainer tubes containing the blood samples were kept overnight and sera then collected in 2-ml cryotubes and stored at – 20 °C for subsequent detection of antibodies against TBPs. The EDTA blood tubes were refrigerated at 4 °C prior to molecular analysis for detection of *T. parva*. Information on animal characteristics, including sex, age and breed, was collected using a questionnaire. Geographic coordinates were collected using an eTrex10 Global Positioning System Device (GARMIN, Olathe, KS, USA).

A second sampling was conducted in each commune to determine the incidence rate of ECF and bovine babesiosis across the country. We had previous collected data on the frequency of TBDs from clinical case records obtained from the respective veterinary services department in each commune. To further understand the effect of husbandry practices on the occurrence of ECF, we conducted a survey across the western zone of the country (Bubanza, Cibitoke and Bujumbura provinces). It had been determined that a total of 378 farms should be sampled to ensure a confidence level of 95% and a margin of error of 5% for a population of 22.000 farms in the western zone. In this study, we randomly selected 395 farms and asked the farmers to complete a questionnaire aimed at eliciting information on their perception of diseases affecting their cattle and husbandry practices, including grazing system, animal health care status and vector control strategies.

### DNA extraction

Genomic DNA was extracted from the EDTA blood samples using the DNeasy Blood & Tissue kit following the manufacturer’s protocol (Qiagen, Hilden, Germany). The quantity and purity of purified DNA samples were measured with the NanoDrop 2000 spectrophotometer (Thermo Fisher Scientific, Waltham, MA, USA). The integrity of total DNA was checked by standard agarose gel electrophoresis. DNA samples were stored at − 20 °C for molecular biology analyses.

### Detection of TBPs

#### *T. parva* detection by p104 gene-based nested PCR

DNA samples were screened for the presence of *T. parva* by a nested PCR using primers derived from the p104 antigen gene, as previously described [14, 15]. This *T. parva-*specific p104 gene-based nested PCR assay is sensitive and specific, with a sensitivity of up to 0.4 parasites/μl of blood, thereby enabling detection of *T. parva* DNA in acute clinical cases as well as from carrier animals [15].

#### Detection of antibodies against TBPs using an enzyme-linked immunosorbent assay

Enzyme-linked immunosorbent assays (ELISA) were used to detect antibodies to *Theileria parva*, *T. mutans*,* Anaplasma marginale* and *Babesia bigemina* in sera according to the protocols provided with the kits [16, 17]. For logistical reasons, 398 samples were selected randomly from the pool of samples and tested for the presence of sera specific antibody for *T. parva*, *A. marginale*,* B. bigemina* and *T. mutans* by ELISA. This sample size ensured a 95% confidence level with a degree of accuracy of 5% in the analysis. Serum samples were assayed for antibodies to *T. parva* by ELISA using the recombinant polymorphic immunodominant molecule, as previously described [16]. Antibodies to *A. marginale, B. bigemina* and *T. mutans* were detected by ELISA using major surface protein 5, the 200-kDa antigen and the 32-kDa antigen, respectively. Optical density readings for test sera were expressed as percentage of positivity (PP). PP values > 20 indicated that an animal was positive for antibodies to *T. parva* and *T. mutans* [16], while PP values > 15 indicated that an animal was positive for antibodies to *A. marginale* and *B. bigemina.*

### Data analysis

Data were recorded in Microsoft Excel (Microsoft Corp., Redmond, WA, USA) and analyzed using R statistical software v3.5 (R Foundation for Statistical Computing, Vienna, Austria). Prevalence of TBPs was calculated, and prevalence at different time points was compared using chi-square. The lower and upper 95% confidence intervals were calculated for binomial proportions. The odds ratio for risk factors (age, sex, breed, grazing system, vector control method, AEZ, animal health care) were estimated together with their confidence intervals. The* P* values for statistical significance were set at 0.05. Spatial distribution of *T. parva* infection was calculated by the inverse distance weighted interpolation technique in QGIS 3.2 software [18]. The distribution and incidence proportion of bovine theileriosis and babesiosis were mapped at the commune level.

## Results

### Demographic characteristic of the study population

A total of 828 cattle blood samples were collected from animals in 24 districts. Approximately 25% of the sampled cattle were between 6 and 12 months old, and the remainder were adults (> 1 year old). About 68% of animals were the local breed (Ankole) and the remainder were Holstein/Sahiwal crosses. Of the 828 cattle sampled, 80% were females.

### Prevalence of *T. parva* infection

The overall prevalence of *T. parva* was 80.55% (667/828 samples) and 86.35% (715/828) by the *T. parva-*specific p104 gene-based nested PCR and polymorphic immunodominant molecule (PIM) ELISA, respectively. The prevalence rate was almost similar across the AEZs, although the Imbo region had a slightly higher prevalence when compared to other AEZs (Table 1). The difference in *T. parva* infection between AEZs were not statistically significant (*χ*^2^  =  2.58, *df*  = 4, * P*  = 0.63).

**Table 1:** *Theileria parva* prevalence based on the *T. parva-*specific p104 gene-based nested PCR in different agro-ecological zones

AEZ	Number of cattle sampled	Number of positive samples	Prevalence (%)	95% CI
Central Highlands	167	131	78.44	71.43–84.42
Congo-Nile Divide	241	193	80.08	74.47–84.93
Depressions	240	194	80.83	75.27–85.61
Imbo	100	86	86.00	77.63–92.13
Slope of CND	80	63	78.75	68.17–87.11
Total	828	667	80.55	77.7–83.1

AEZ, Agro-ecological zone; CI, confidence interval; CND, Congo–Nile Divide

The prevalence rate across the communes is summarized in Table 2. The commune of Cankuzo exhibited a higher seroprevalence rate; however, the infection rate was low when the PCR assay was used. The communes of Makebuko, Muramvya, Makamba and Mutimbuzi had the highest prevalence rate (> 85%) by PCR. The Pearson Chi-squared test showed that the *T. parva* infection rate was associated with the commune (*χ*^2^ = 23.34, *df* = 9, *P* = 0.005).

**Table 2:** Prevalence in different communes based on the enzyme-linked immunosorbent assay and p104 gene-based nested PCR

Commune	Number of cattle sampled	*T. parva-*specific p104 gene-based nested PCR			PIM ELISA		
		Number of positive samples	Prevalence (%)	95% CI	Number of positive samples	Prevalence (%)	95% CI
Makebuko	80	72	90.0	81.2–95.6	74	92.5	84.4–97.2
Muramvya	75	65	86.6	76.8–93.4	65	86.6	76.8–93.4
Cankuzo	87	59	67.8	56.9–77.4	81	93.1	85.6–97.4
Gisozi	82	61	74.4	63.6–83.4	67	81.7	71.6–89.4
Giharo	80	59	73.7	62.7–83.0	73	91.2	82.7–96.4
Makamba	80	68	85.0	75.3–92.0	70	87.5	78.2–93.8
Mugongomanga	84	67	79.8	69.6–87.7	73	86.9	77.7–93.2
Kirundo	80	67	83.7	73.8–91.1	62	77.5	66.7–86.0
Rugombo	80	63	78.7	68.2–87.1	67	83.7	73.8–91.0
Mutimbuzi	100	86	86.0	77.6–92.1	83	83.0	74.1–89.7
Total	828	667	80.55	77.7–83.1	715	86.35	83.8–88.6

ELISA, Enzyme-linked immunosorbent assay PIM, polymorphic immunodominant molecule

The prevalence rates according to sex, breed and age are summarized in Table 3. The probability of *T. parva* infection in male cattle was lower than than in female cattle, while calves were at higher risk of being infected than adult cattle. The local Ankole breed and the exotic/crossbreed’ cattle were at the same risk of being infected by *T. parva*. The logit regression analysis showed that the association between age and *T. parva* infection was statistically significant (*P* = 0.01). However, the difference in *T. parva* infection was not statistically significant with regard to the breed and sex of cattle (*P* > 0.05).

**Table 3:** PCR-based *T. parva* prevalence and infection risk according to age, sex, and breed of cattle sampled

Animal characteristics	Number of cattle sampled	Number of positive samples	Prevalence (%)	Odds ratio	95% CI	*P* value
Age						
Adult (> 12 months)	626	492	78.5	Ref		
Calves (6–12 months)	202	175	86.6	1.77	1.13–2.76	0.01*
Sex						
Female	675	552	81.7	Ref		
Male	153	115	75.2	0.67	0.45–1.02	0.07
Breed						
Crossbreed cattle	262	212	80.9	Ref		
Local Ankole breed	566	455	80.4	0.97	0.67–1.40	0.86

*Significant difference at *P* < 0.05

### Spatial distribution of *T. parva* infection according to the p104 gene-based nested PCR

**Fig. 1:**
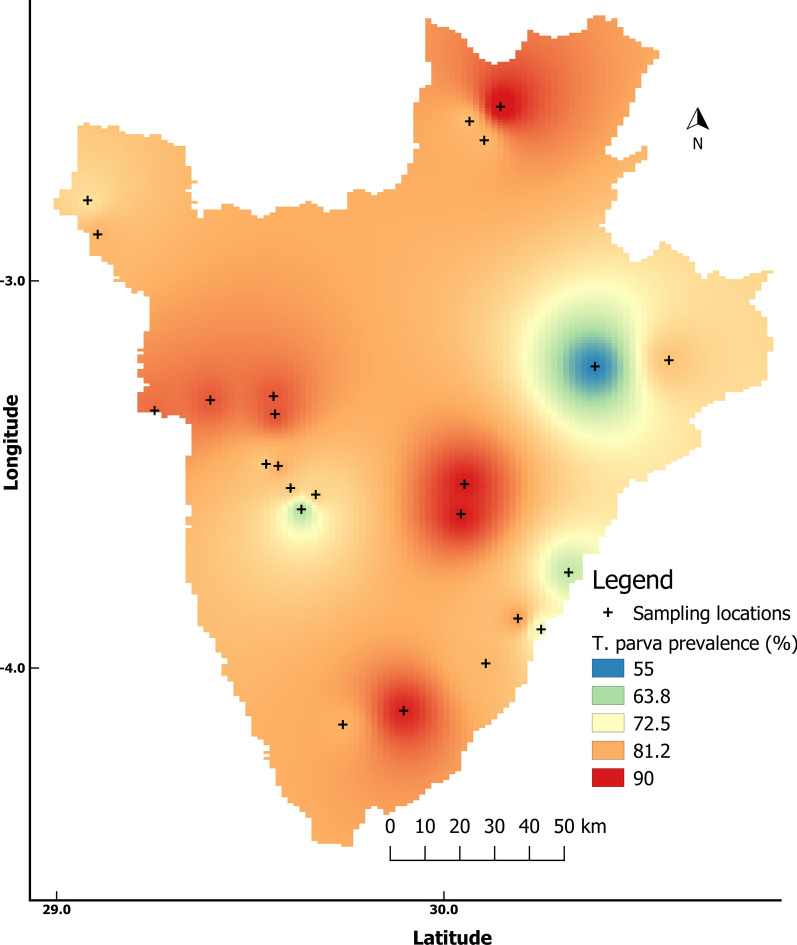
Spatial distribution of *Theileria parva* infection in Burundi

The number of cattle sampled per district and the association between the 24 districts and the communes and AEZs are summarized in Table 4. The prevalence of *T. parva* at the district level was variable, ranging from 55 to and 92%. The districts of Nyabibugu (Kirundo), Makebuko and Buhoro in Makebuko commune had the highest prevalence rate (≥ 90%), and Cankuzo, Gisozi and Rubanga districts had a relatively lower prevalence rate (< 70%). The districts located in the eastern depressions showed a prevalence rate of between 70 and 80% (Fig. 1).

**Table 4:** District level *T. parva* prevalence according to the p104 gene-based nested PCR

District	Commune	AEZ	Coordinate (*x*;* y*)	Number of cattle sampled	Number of positive samples	Prevalence (%)	95% CI
Cankuzo	Cankuzo	Highlands	30.39; − 3.22	40	22	55	39.8–69.2
Nyakerera	Cankuzo	Highlands	30.58; − 3.20	47	37	78.7	65.0–88.0
Buhogo	Giharo	Depressions	30.11; − 3.99	15	12	80	54.8–92.9
Mwebeya	Giharo	Depressions	30.25; − 3.91	27	19	70.3	51.5–84.1
Rubanga	Giharo	Depressions	30.32; − 3.75	21	14	66.6	45.3–82.8
Shasha	Giharo	Depressions	30.19; − 3.87	17	14	82.3	58.9–93.8
Nyarumanga	Gisozi	CND	29.60; − 3.53	42	33	78.5	64.0–88.2
Gisozi	Gisozi	CND	29.63; − 3.59	22	14	63.6	42.9–80.2
Rutongati	Gisozi	CND	29.67; − 3.55	18	14	77.7	54.7–90.9
Karehe	Kirundo	Depressions	30.07; − 2.59	29	23	79.3	61.6–90.1
Nyabibugu	Kirundo	Depressions	30.15; − 2.55	26	24	92.3	75.8–97.8
Gasura	Kirundo	Depressions	30.10; − 2.64	25	20	80	60.8–91.1
Makamba	Makamba	Depressions	29.74; − 4.15	42	34	80.9	66.6–90.0
Mahinda	Makamba	Depressions	29.90; − 4.11	38	34	89.4	75.8–95.8
Buhoro	Makebuko	Highlands	30.05; −3.53	20	18	90	69.8–97.2
Makebuko	Makebuko	Highlands	30.04; − 3.60	60	54	90	79.8–95.3
Ijenda	Mugongomanga	CND	29.57; − 3.48	43	35	81.4	67.3–90.2
Rwibaga	Mugongomanga	CND	29.54; − 3.47	41	32	78	63.2–87.9
Mpehe	Muramvya	CND	29.56; − 3.30	22	19	86.3	66.6–95.2
Muramvya	Muramvya	CND	29.56; − 3.34	53	46	86.7	75.1–93.4
Rubirizi	Mutimbuzi	Imbo	29.40; − 3.31	80	69	86.2	77.0–92.1
Gatumba	Mutimbuzi	Imbo	29.25; − 3.33	20	17	85	63.9–94.7
Kagazi	Rugombo	Slope of CND	29.08; − 2.79	29	22	75.8	57.8–87.7
Rugombo	Rugombo	Slope of CND	29.11; − 2.88	51	41	80.3	67.5–88.9

*x*, Longitude; *y*, latitude

### Seroprevalence of other TBPs and co-infections in cattle

The mean seroprevalence of *T*. *mutans*, *B*. *bigemina*, and *A. marginale* in the cattle sampled across all communes was 63.8% (254/398), 63.5% (253/398) and 76.3% (304/398), respectively (Table 5). *Babesia bigemina* prevalence was highest (90%) in the Rugombo and Giharo communes and lowest in Kirundo and Makebuko communes (32.5 and 35.0%, respectively). *Anaplasma marginale* prevalence was highest in Cankuzo commune (97.5%) and lowest (50.0%) in Mugongomanga commune. The highest and lowest prevalence of *T. mutans* was recorded in Mutimbuziat commune (92.1%) and Makebuko and Kirundo communes (30%), respectively.

**Table 5:** Seroprevalence of bovine tick-borne pathogens

Commune	Number of cattle sampled	*Babesia bigemina*			*Anaplasma marginale*			*Theileria mutans*		
		Number of positive samples	Prevalence (%)	95% CI	Number of positive samples	Prevalence (%)	95% CI	Number of positive samples	Prevalence (%)	95% CI
Giharo	40	36	90.0	76.9–96.0	38	95.0	83.5–98.6	29	72.5	57.1–83.9
Makamba	40	28	70.0	54.6–81.9	38	95.0	83.5–98.6	31	77.5	62.5–87.7
Kirundo	40	13	32.5	20.1–47.9	29	72.5	57.1–83.9	12	30.0	18.0–45.4
Mugongomanga	40	30	75.0	59.8–85.8	20	50.0	35.2–64.8	19	47.5	32.9–62.5
Muramvya	40	18	45.0	30.7–60.2	24	60.0	44.5–73.6	23	57.5	42.1–71.4
Mutimbuzi	38	27	71.0	55.2–82.9	26	68.4	52.5–80.9	35	92.1	79.2–97.2
Rugombo	40	36	90.0	76.9–96.0	38	95.0	83.5–98.6	28	70.0	54.6–81.9
Cankuzo	40	28	70.0	54.6–81.9	39	97.5	87.1–99.5	31	77.5	62.5–87.7
Makebuko	40	14	35.0	22.1–50.5	30	75.0	59.8–85.8	12	30.0	18.0–45.4
Gisozi	40	23	57.5	42.1–71.5	22	55.0	39.8–69.2	34	85.0	70.9–92.9
Total	398	253	63.5	58.7–68.1	304	76.3	71.9–80.2	254	63.8	58.9–68.3

Mixed infections were detected in 82.91% (330/398) of cattle, with 11 different combinations of TBP species. Co-infections involving four pathogens were observed in 40% (160/398) of cattle, while 30% (118/398) of the animals had three pathogens, 13% (52/398) had two pathogens and 11% (45/398) were infected by a single pathogen. *Theileria parva* was detected as a single infection in 18 samples (4.5%), *A. marginale* in 20 samples (5.0%), *B. bigemina* in two samples (1.2%) and *T. mutans* in five samples (1.2%). Co-infections were detected as follows: *T. parva* and *T. mutans*, in 2.2% of samples; *T. parva* and *B. bigemina*, in 2.5% of samples; *T. parva* and *A. marginale* in 5.5% of samples; and *T. mutans* and *B. bigemina*, in 0.25% of samples; three samples had co-infections with *T. mutans* and *A. marginale* (0.7%) and seven samples (1.75%) had co-infections with *B. bigemina* and *A. marginale*. *Theileria parva*, *T. mutans* and *B. bigemina* mixed infections were found in 6.5% of samples; *T. parva, T. mutans* and *A. marginale* in 11.3%; *T. mutans*, *B. bigemina* and *A. marginale* in 1.2%; and *T. parva*, *B. bigemina* and *A. marginale* in 10.5%. Co-infection involving four pathogens, namely *A. marginale*, *B. bigemina*, *T. mutans* and *T. parva*, was the most frequent combination, accounting for 40% of infections and detected in 160 samples.

### Incidence of clinical ECF and babesiosis

#### East Coast fever

**Fig. 2:**
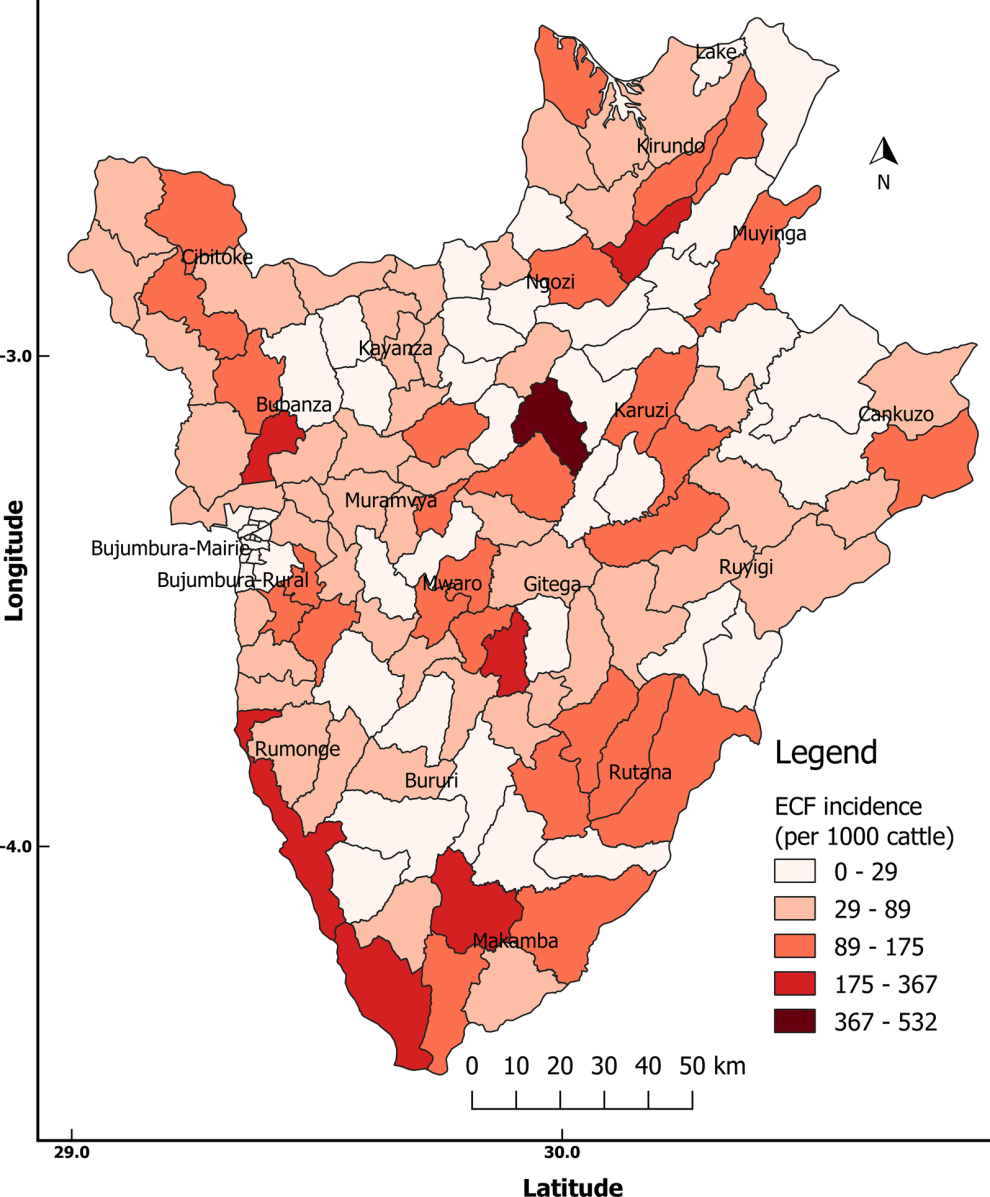
Incidence proportion of East Coast fever (*ECF*) in Burundi 2017

Our results showed that the overall incidence of ECF in Burundi in 2017 was 74.5 cases per 1000 cattle. The highest incidence (367–532 cases per 1000 cattle) was recorded in Makamba and Gihogazi (Karusi) communes, whereas the lowest incidence (< 36.0 cases per 1000 cattle) was registered in Tangara and Mwumba communes in Ngozi Province and Gitaramuka and Nyabikere communes in Karuzi Province (Fig. 2).

#### Bovine babesiosis

**Fig. 3:**
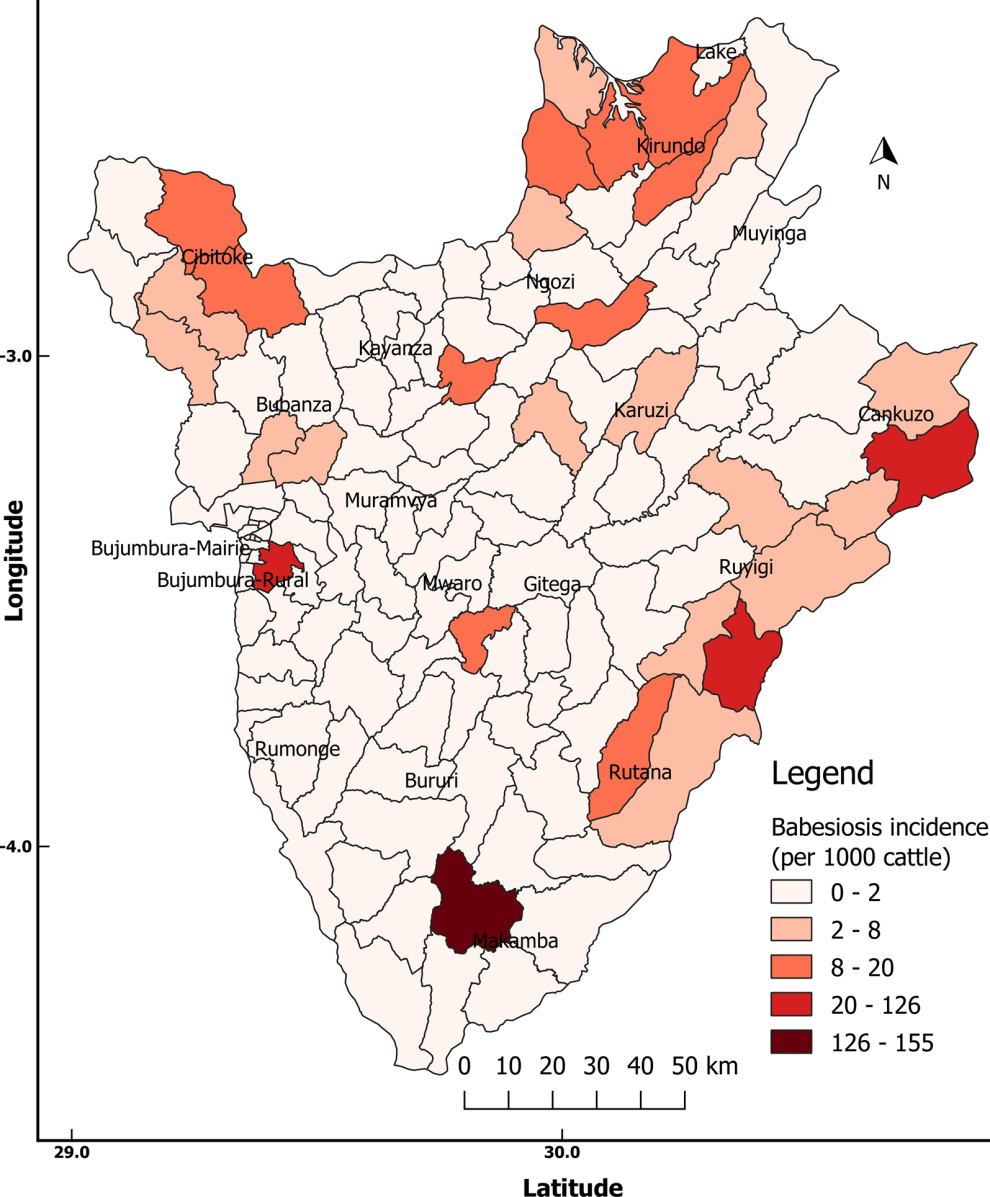
Incidence proportion of bovine babesiosis in Burundi in 2017

The overall incidence of bovine babesiosis in Burundi was 5.13 cases per 1000 cattle in 2017. The highest incidence (126.0–155.2 cases per 1000 cattle) was recorded in Kanyosha (Bujumbura Rural) and Makamba communes (Additional file 1: Table S1). The lowest incidence (< 2.0 cases per 1000 cattle) was recorded in both Muramvya and Gitega communes (Fig. 3).

### Influence of animal husbandry practices on ECF occurrence

A total of 395 farmers in 24 districts located across the western zone (Cibitoke, Bubanza and Bujumbura provinces) participated in the survey. Approximately 75% of the farms were in the Imbo AEZ, while the remaining farms were within the CND and the slope of Mumirwa AEZs. About 55% of farmers reared cattle under a zero-grazing system, while 35% managed their animals under an open grazing system; the remainder alternated between these two systems. The majority (96%) of the farmers applied acaricide for controlling tick vectors while the remainder plucked ticks off cattle (3%) or practiced no tick control at all(1%). Only 40% of farmers used the services of a trained animal health expert.

The proportion of ECF in farms and the risk of occurrence according to AEZ, grazing system and animal health care are summarized in Table 6. The CND had the high rate of ECF of all AEZs. The difference in ECF proportion was statistically significant between AEZs (*χ*^2^ = 30.69, *df* = 2, *P* < 0.001). The open grazing system was associated with a higher risk of ECF occurrence in comparison to other grazing systems, and the difference was statistically significant (*χ*^2^ = 15.48, *df* = 2, *P* < 0.001). Regarding animal health care, farmers who were more affected by ECF tended to hire the services of an animal health professional as compared to farmers who were less affected The animal health care in farms was statistically associated with the proportion of ECF (*χ*^2^ = 12.61, *df* = 1, *P* < 0.001). The methods used by farmers to control ticks were not statistically associated with ECF occurrence (*χ*^2^ = 0.98, *df* = 2, *P* = 0.61).

**Table 6:** Occurrence and risk of East Coast fever according to ago-ecological zone, grazing and animal health care in the western zone of Burundi

Husbandry practices	Number of farms	ECF occurrence	Proportion of ECF (%)	Odds ratio	95% CI
Agro-ecological zone					
CND	41	33	80.48	Reference	
Imbo	295	189	64.06	0.26*	0.08–0.73
Slope of Mumirwa	59	18	30.50	0.07*	0.02–0.24
Grazing system					
Mixed (zero and open)	37	26	70.27	Reference	
Open grazing	141	101	71.63	2.21*	0.83–5.90
Zero grazing	217	113	52.07	0.75*	0.28–1.96
Animal health care					
No veterinary care	232	124	53.44	Reference	
Care by veterinary professional	163	116	71.16	3.48*	2.14–5.76

ECF, East Coast fever

*Significant difference compared to Reference at *P* < 0.00

## Discussion

The prevalence of *T. parva* infection and incidence of the disease recorded in this study suggests endemic stability of ECF across AEZs throughout Burundi. We found a high prevalence of *T. parva* infection (> 80%) combined with a low incidence rate (< 7.5%). The high rate of *T. parva* prevalence across all of the AEZs of Burundi is likely to be associated with the distribution and abundance of the main tick vector of *T. parva*, *Rhipicephalus appendiculatus*, together with the uncontrolled movement of cattle in Burundi [4]. A high prevalence of the parasite was observed in the western and south-eastern regions of the country, neighboring the Democratic Republic of Congo (DRC) and Tanzania, respectively, where trans-border movement and trade of animals is common [2]. Thousands of exotic and crossbreed cattle are imported into Burundi from neighboring countries, particularly from Tanzania and Uganda, to improve animal productivity. However, the animals are not screened for *T. parva* before being allowed into the country. *Rhipicephalus appendiculatus* abundance is low in the area where the altitude exceeds 2300 m a.s.l. and/or there is a monomodal pattern of rainfall [4]. A relatively lower prevalence (< 65%) was found in the eastern and northern parts of the country, characterized by a low level of humidity. Likewise, the Gisozi district, located at the highest altitude (> 2000 m a.s.l.) of the CND presented a lower prevalence. Although different microclimatic areas and vegetation types are found across the AEZs of Burundi, high vegetation coverage, combined with temperatures varying between 15 °C and 24 °C and rainfall between 800 and 1600 mm, create a tropical humid climate across most areas that is suitable for *R. appendiculatus* survival and development [1].

Studies on the spatial distribution of *T. parva* infection in countries where it occurs have shown that spatial distribution is associated with vector suitability, host genetics, farmer management practices and sociodemographic processes [19–21]. This study found ECF occurrence in farms to be associated with AEZ and husbandry practices (grazing system and animal health care). In Tanzania, Kazungu et al. [22] reported that cattle reared under a system of regular tick control had significantly lower antibody levels compared to those managed under irregular tick control [22]. Acaricides have been extensively used by farmers in Burundi for tick control [23]. However, the high cost of these products and the lack of financial means limit the use of acaricides in cattle kept under semi-intensive production characterized by mixed crop–livestock systems with a continuous tick challenge. High seroprevalence rates (> 75%) have been reported in Kenya and were associated with grazing systems [21]. Regardless of cattle breed, animals reared under open grazing systems exhibited a high seroprevalence rate, which was likely due to continuous high levels of tick challenge. A previous study in Burundi showed that *R. appendiculatus* is the most prevalent tick vector and that animals may be encountering this vector year round, with high levels of tick challenge, particularly in the free-range grazing systems [24].

The high rate of pathogen and antibody prevalence associated with a low incidence of clinical cases suggests that an endemic status of ECF exists in Burundi. Endemic stability is likely to occur when more than 70% of the population become infected, as indicated by a high antibody prevalence (> 70%) and little clinical disease occurs [25, 26]. The endemic status of ECF in Burundi is probably due to the presence of the resistant local Ankole cattle, suitable ecological factors for the vector and a regular transmission of the parasite.

### Performance of serological and molecular tools in *T. parva* detection

Molecular and serological tools are currently being used for *T. parva* detection. The ELISA has the advantages of being inexpensive and rapid, and can be used as a high-throughput screening method [27]. In addition to ELISA, many national laboratories in sub-Saharan Africa are now equipped with molecular diagnostic platforms, such as PCR. In our study, the level of concordance between the serological and molecular methods in terms of the detection of *T. parva* infection was assessed. The PIM antigen-based ELISA assay showed a similar prevalence rate as the molecular p104 diagnostic nested PCR assay. The serological method allows the detection of antibodies, thus identifying both current and past exposure, whereas the p104 gene-based PCR detection system mostly reflects current infection. The high rate of concordance between the two detection methods could be explained by a continuous tick challenge and transmission with continued presence of the parasites on cattle leading to boosting of pathogen-specific antibody levels. This situation provides a high prevalence of parasites, eliciting a high serological prevalence. In a different tick challenge setting, the agreement between the two assays may be different. Gachohi et al. [21] reported that agreement between pathogen prevalence and antibodies could depend on the frequency of acaricide application, age of animals and the resistance of the hosts. Kazungu et al. [22] observed a low *T*. *parva* parasite prevalence associated with high antibody levels of *T*. *parva* infection in Tanzania. These authors suggested that this was possible in animals that could have recovered from previous *T*. *parva* infection, hence the low parasitaemia was not detectable by molecular tools even though high antibodies levels were still present in cattle.

### *T. parva* risk infection by age, sex and breed

Animal characteristics, including age, sex and breed, were recorded with the aim to identify the risk factors for *T. parva* infection in cattle in Burundi. Local Ankole cattle and crossbred cattle exhibited the same risk of infection by *T. parva* parasites. Similar results were found in Uganda and Kenya where no significant association was detected between cattle breed and prevalence rate of *T. parva* [21, 28]. In contrast, Simuunza et al. [41] reported that cattle breed was associated with *T. parva* infection risk in Zambia. In recent decades, the local Ankole cattle have been crossed with the exotic taurine and indicine cattle breeds (Friesian, Brown Swiss, Sahiwal, etc.) to increase milk and meat production in Burundi [11, 12]. Hence, crossbred cattle represent a significant proportion of cattle in Burundi. However, they still maintain some level of resistance against TBDs.

A higher risk of infection for female cattle was identified in the present study and could be possibly be explained by the fourfold higher number of female cattle relative to male cattle in the Burundi cattle population. Also, male cattle are provided with better health care due to their higher value, as they are used by farmers for reproduction and sold for meat, whereas females are kept for dairy. In Uganda, Muhanguzi et al. [28] found that age group and sex were not associated with the prevalence of ECF. Our study showed that calves had a higher chance of being infected compared to adults. Adult cattle that were infected as calves are resistant to re-infection, which could explain the high risk of infection for calves compared to adult animals [21].

### Seroprevalence of multiple co-infections indicates the overlapping distribution of tick vectors in Burundi

Tick-borne diseases, including bovine babesiosis, anaplasmosis, cowdriosis and theileriosis, have been reported in Burundi where tick vectors are widely distributed across the country [24, 29]. Ixodid ticks can transmit a large number of pathogens, including species of genera *Theileria*, *Babesia* and *Anaplasma* [30]. Parasite co-infections have an impact on cattle survival, treatment and clinical outcomes [31–33]. In Burundi, recent information on the prevalence of single or mixed infections with *Anaplasma*,* Theileria* and *Babesia* species in cattle is very limited.

In this study we found a high overall mean seroprevalence rate of *T. mutans*, *B. bigemina* and *A. marginale* (> 63%). The occurrence of the tick *Rhipicephalus* (*Boophilus*)* decoloratus*, which transmits *B. bigemina* and *A. marginale*, has previously been reported in Burundi [24]. *Rhipicephalus *(*Boophilus*)* microplus*, a sister species which transmits *Babesia bovis* in addition to *B. bigemina* and *A. marginale*, has not been reported in Burundi. However, recent studies have reported the expansion of the invasive *R. microplus* in Africa [34], with reports of it spreading rapidly across West and Central Africa [35]. This tick has recently been reported in eastern Uganda [36], confirmed to be present in Kenya and is known to occur in Tanzania and Zambia, where it has largely replaced the indigenous *R. decoloratus* [37, 38]. The wide occurrence and distribution of *R. decoloratus* ticks across the country explains the observed high prevalence of *B. bigemina* and *A. marginale* in different communes within Burundi [39]. The highest prevalence rates of *B. bigemina* and *A*. *marginale* antibodies were found in Cibitoke and Cankuzo communes located in the western region and the eastern depression, respectively. Similar findings were reported in other sub-Saharan Africa countries (Kenya, Sudan) which also exhibited a high prevalence rate of anaplasmosis and babesiosis [39, 40]. The high seroprevalence of *Babesia* species (63%) and the low incidence of disease (0.5%) suggest that babesiosis may also be endemic in Burundi.

We observed a high number of mixed parasite infections, with 40% of cattle found to be infected with four different parasites, namely *B. bigemina*,* T. mutans*,* T. parva* and *A. marginale*. Mixed infections consisting of two or more infections were detected in 83.1% of cattle sampled, with 11 different combinations of parasite species identified. These results are most likely due to the overlapping distribution of different tick species, such as *R. appendiculatus*,* R. decoloratus* and *Amblyomma variegatum*, across the country where these vectors share similar habitats and ecological niches [4]. It is probable that many cattle are infested with multiple tick species which may transmit several pathogens to individual animals [41]. *Theileria mutans* infection has been reported to be associated with a decrease in the occurrence of clinical ECF, following subsequent infection by *T. parva* [31]*.* Cattle co-infected with *T. parva* and *T. mutans* have reduced morbidity relative to those infected by *T. parva* alone. Moreover, co-infection with less pathogenic *Theileria* species (specifically *T. mutans* or *T. velifera*) results in a significant reduction in mortality due to *T. parva* infection [32]. However, the mechanism by which *T. parva* and *T. mutans* infections interact to result in a reduced mortality rate is still unclear. In the present study we found a high level of co-infection between *T. parva* and *T. mutans*, ranging from 48 to 100% within different communes; however, it was not possible to relate this to mortality levels in cattle since this was a horizontal survey.

### Strategic immunization and chemotherapy should play a key role in ECF control in Burundi

Control strategies for ECF, either through tick vector control, immunization or disease treatment, should be based on knowledge of the epidemiology of the disease. The results of the present study suggest an endemic stability of *T. parva* and a high prevalence rate of other TBPs across the different AEZs of Burundi.

The need to adopt ITM immunization when theileriosis incidence is above 5% and disease treatment when the incidence is between 1.5 and 5% has been suggested as the most economic strategy [42]. Although the prevalence of ECF (pathogen and antibodies) is very high (> 80%) in Burundi, the incidence of the disease was estimated to be 7.45%. Adoption of ITM immunization could be the most economical approach to reduce ECF-induced mortality while concomitantly maintaining endemic stability status through limited control of tick vectors, rather than complete elimination. However, the less productive local Ankole cattle have a lower susceptibility to clinical ECF, with a mortality rate of less than 10% for calves. In contrast, improved exotic and crossbreed cattle, which are high producers, are most affected by ECF, with an estimated annual mortality rate of 40% [43]. Immunization using the ITM would target exotic and crossbreed animals reared under semi-intensive and intensive livestock systems. The commercially available live vaccine, called the “Muguga Cocktail,” is currently being used in Burundi’s neighboring countries [7]. However, it should be noted that to date adoption has been higher among extensive pastoralist systems than among keepers of improved breeds [7].

A useful additional level of control would be for animal health services to adopt a screening strategy for TBPs of cattle imported from neighboring countries to limit introduction of new *T. parva* and other TBP isolates.

## Conclusion

A high prevalence of TBPs, particularly *T. parva*, was observed in Burundi. The overlapping distribution of multiple disease-transmitting tick vectors is likely to be responsible for the high infection rates observed. Further studies are needed to assess the population structure of *T. parva* in Burundi in comparison with those found in countries where the Muguga Cocktail live vaccine has been successfully deployed.

## Supplementary Information

**Additional file 1: Table S1.** Number of clinical cases of ECF and bovine babesiosis per 1000 cattle and per commune in 2017.

## Data Availability

The data that support the findings of this study are presented in the article and additional files.

## Abbreviations

AEZ: Agro-Ecological Zone
ECF: East Coast fever
ELISA: Enzyme-linked immunosorbent assay
ITM: Infection treatment method
MSP5: Major surface protein 5
PIM: Polymorphic immunodominant molecule
TBD: Tick-borne disease
TBP: Tick-borne pathogen

## References

[1] Hatungumukama G, Hornick JL, Detilleux J (2007). Aspects zootechniques de l’élevage bovin laitier au Burundi: Présent et futur. Ann Méd Vét..

[2] Nkurunziza S (2016). Report of the national livestock census.

[3] Nsanganiyumwami D (2017). Animal health reports 2012–2017.

[4] Sutherst RW, Bourne AS, Floyd R, Museru B (1986). Lutte contre les tiques et les maladies parasitaires des bovins: les tiques des bovins, leur identité, ecologie et relations avec les maladies; la politique de lutte contre celles ci.

[5] Irvin AD, Cunningham MP, Young AS (editors). Advances in the control of theileriosis. Proceedings of an International Conference held at the International Laboratory for Research on Animal Diseases in Nairobi, 9–13th February, 1981. Current topics in veterinary medicine and animal science series, vol 14. Dordrecht: Springer. p. 227–37.

[6] Radley DE, Brown CGD, Cunningham MP, Kimber CD, Musisi FL, Payne RC (1975). East coast fever: 3. Chemoprophylactic immunization of cattle using oxytetracycline and a combination of theilerial strains. Vet Parasitol.

[7] Di Giulio G, Lynen G, Morzaria S, Oura C, Bishop R (2009). Live immunization against East Coast fever—current status. Trends Parasitol..

[8] Tama E, Banuma A (1987). L’immunisation des veaux contre la theileriose au Burundi.

[9] Kiltz HH, Humke R (1986). Bovine theileriosis in Burundi: chemotherapy with halofuginone lactate. Trop Anim Health Prod..

[10] HarvestChoice; International Food Policy Research Institute (IFPRI). Agro-Ecological Zones for Africa South of the Sahara. Harvard Dataverse; 2015. https://dataverse.harvard.edu/dataset.xhtml?persistentId=doi:10.7910/DVN/M7XIUB. Accessed 21 May 2020.

[11] Manirakiza J, Hatungumukama G, Th Evenon S, Gautier M, Besbes B, Flori L (2017). Effect of genetic European taurine ancestry on milk yield of Ankole-Holstein crossbred dairy cattle in mixed smallholders system of Burundi highlands. Anim Genet..

[12] Wurzinger M, Ndumu D, Baumung R, Drucker A, Okeyo AM, Semambo DK (2006). Comparison of production systems and selection criteria of Ankole cattle by breeders in Burundi, Rwanda, Tanzania and Uganda. Trop Anim Health Prod..

[13] Toye P, Handel I, Gray J, Kiara H, Thumbi S, Jennings A (2013). Maternal antibody uptake, duration and influence on survival and growth rate in a cohort of indigenous calves in a smallholder farming system in western Kenya. Vet Immunol Immunopathol..

[14] Skilton RA, Bishop RP, Katende JM, Mwaura S, Morzaria SP (2002). The persistence of *Theileria parva* infection in cattle immunized using two stocks which differ in their ability to induce a carrier state: Analysis using a novel blood spot PCR assay. Parasitology.

[15] Odongo DO, Sunter JD, Kiara HK, Skilton RA, Bishop RP (2010). A nested PCR assay exhibits enhanced sensitivity for detection of *Theileria parva* infections in bovine blood samples from carrier animals. Parasitol Res..

[16] Katende J, Morzaria S, Toye P, Skilton R, Nene V, Nkonge C (1998). An enzyme-linked immunosorbent assay for detection of *Theileria parva* antibodies in cattle using a recombinant polymorphic immunodominant molecule. Parasitol Res..

[17] Morzaria SP (1999). Development of sero-diagnostic and molecular tools for the control of important tick-borne pathogens of cattle in Africa. Parassitologia..

[18] Pullan RL, Sturrock HJW, Soares Magalhães RJ, Clements ACA, Brooker SJ (2012). Spatial parasite ecology and epidemiology: a review of methods and applications. Parasitology.

[19] Gitau GK, McDermott JJ, Katende JM, O’Callaghan CJ, Brown RN, Perry BD (2000). Differences in the epidemiology of theileriosis on smallholder dairy farms in contrasting agro-ecological and grazing strata of highland Kenya. Epidemiol Infect..

[20] Weny G, Okwee-Acai J, Okech SG, Tumwine G, Ndyanabo S, Abigaba S (2017). Prevalence and risk factors associated with hemoparasites in cattle and goats at the edge of Kibale National Park, Western Uganda. J Parasitol..

[21] Gachohi J, Skilton R, Hansen F, Ngumi P, Kitala P (2012). Epidemiology of East Coast fever (*Theileria parva* infection) in Kenya: past, present and the future. Parasites Vectors..

[22] Kazungu YEM, Mwega E, Kimera SI, Gwakisa P (2015). Seroprevalence and carrier state of *Theileria parva* in cattle under two tick control regimes in small-holder farming systems of Tanzania. Livest Res Rural Dev..

[23] Moran MC, Nigarura G (1990). Strategic tick control in Burundi. Parassitologia..

[24] Moran MC, Nigarura G, Pegram RG (1996). An assessment of host resistance to ticks on cross-bred cattle in Burundi. Med Vet Entomol..

[25] Norval RAI, Perry BD, Young AS (1992). The epidemiology of theileriosis in Africa.

[26] Perry BD, Young AS (1995). The past and future roles of epidemiology and economics in the control of tick-borne diseases of livestock in Africa: the case of theileriosis. Prev Vet Med..

[27] Mans BJ, Pienaar R, Latif AA (2015). A review of *Theileria* diagnostics and epidemiology. Int J Parasitol Parasites Wildl..

[28] Muhanguzi D, Picozzi K, Hatendorf J, Thrusfield M, Welburn SC, Kabasa JD (2014). Prevalence and spatial distribution of Theileria parva in cattle under crop-livestock farming systems in Tororo District, Eastern Uganda. Parasites Vectors..

[29] De Meneghi D, Stachurski F, Adakal H (2016). Experiences in tick control by Acaricide in the Traditional Cattle Sector in Zambia and Burkina Faso: possible environmental and public health implications. Front Public Health..

[30] Moutailler S, Valiente Moro C, Vaumourin E, Michelet L, Tran FH, Devillers E (2016). Co-infection of ticks: the rule rather than the exception. PLoS Negl Trop Dis..

[31] Thumbi SM, de Bronsvoort BMC, Poole EJ, Kiara H, Toye PG, Mbole-Kariuki MN (2014). Parasite co-infections and their impact on survival of indigenous cattle. PLoS ONE.

[32] Woolhouse MEJ, Thumbi SM, Jennings A, Chase-Topping M, Callaby R, Kiara H (2015). Co-infections determine patterns of mortality in a population exposed to parasite infection. Sci Adv..

[33] Van Wyk IC, Goddard A, de Bronsvoort BMC, Coetzer JAW, Handel IG, Hanotte O (2014). The impact of co-infections on the haematological profile of East African Short-horn Zebu calves. Parasitology.

[34] Madder M, Thys E, Achi L, Touré A, De Deken R (2011). *Rhipicephalus (Boophilus) microplus*: a most successful invasive tick species in West-Africa. Exp Appl Acarol..

[35] Silatsa BA, Kuiate JR, Njiokou F, Simo G, Feussom JMK, Tunrayo A (2019). A countrywide molecular survey leads to a seminal identification of the invasive cattle tick *Rhipicephalus (Boophilus) microplus* in Cameroon, a decade after it was reported in Cote d’Ivoire. Ticks Tick Borne Dis..

[36] Muhanguzi D, Byaruhanga J, Amanyire W, Ndekezi C, Ochwo S, Nkamwesiga J (2020). Invasive cattle ticks in East Africa: Morphological and molecular confirmation of the presence of *Rhipicephalus microplus* in south-eastern Uganda. Parasit Vectors..

[37] Jongejan F, Lemche J, Mwase ET, Kafunda MM (1986). Bovine babesiosis (*Babesia bovis* infection) in Zambia. Vet Q..

[38] Lynen G, Zeman P, Bakuname C, Di Giulio G, Mtui P, Sanka P (2008). Shifts in the distributional ranges of Boophilus ticks in Tanzania: evidence that a parapatric boundary between Boophilus microplus and *B. decoloratus* follows climate gradients. Exp Appl Acarol..

[39] Kanduma EG, Emery D, Githaka NW, Nguu EK, Bishop RP, Šlapeta J (2020). Molecular evidence confirms occurrence of *Rhipicephalus microplus* Clade A in Kenya and sub-Saharan Africa. Parasites Vectors..

[40] Salih DA, Abdel Rahman MB, Mohammed AS, Ahmed R, Kamal S, El Hussein AM (2009). Seroprevalence of tick-borne diseases among cattle in the Sudan. Parasitol Res..

[41] Wesonga FD, Gachohi JM, Kitala PM, Gathuma JM, Njenga MJ (2017). Seroprevalence of Anaplasma marginale and Babesia bigemina infections and associated risk factors in Machakos County, Kenya. Trop Anim Health Prod..

[42] Simuunza M, Weir W, Courcier E, Tait A, Shiels B (2011). Epidemiological analysis of tick-borne diseases in Zambia. Vet Parasitol..

[43] D’haese L, Penne K, Elyn R (1999). Economics of theileriosis control in Zambia. Trop Med Int Health..

